# Perceptions of the Doctor-Patient Relationship Among Patients in a Private, Secondary-Level Hospital in Southern India

**DOI:** 10.3389/fpubh.2021.768705

**Published:** 2022-04-06

**Authors:** Pooja Gala, Veena Sriram, Chitra Kotian, Kirthinath Ballala, Rajesh Vedanthan, Emily Perish, Shashikiran Umakanth, David Meltzer

**Affiliations:** ^1^Department of Population Health, NYU Grossman School of Medicine, New York, NY, United States; ^2^Department of Medicine, NYU Grossman School of Medicine, New York, NY, United States; ^3^School of Population and Public Health, School of Public Policy and Global Affairs, University of British Columbia, Vancouver, BC, Canada; ^4^Department of Medicine, Dr. TMA Pai Hospital (Udupi), Melaka Manipal Medical College, Manipal Academy of Higher Education, Manipal, India; ^5^Department of Community Medicine, Kasturba Medical College, Manipal Academy of Higher Education, Manipal, India; ^6^Department of Medicine, University of Chicago, Chicago, IL, United States

**Keywords:** non-communicable diseases, doctor-patient relationship, quality of care, low- and middle-income countries, provider trust

## Abstract

**Introduction:**

An epidemic of non-communicable diseases (NCDs) in India is fueling a growing demand for primary care and hospitalization services. Difficulties in coordinating inpatient and outpatient care create significant barriers to providing high-quality medical care. In this paper, we describe patient experiences, perceptions, and expectations of doctor-patient relationships in a secondary-level private hospital in Karnataka, India.

**Methods:**

We conducted a cross-sectional, mixed-method needs assessment with surveys and in-depth interviews at Dr. TMA Pai Hospital (TMAPH), a secondary-level, private sector hospital in Karnataka, India. Inclusion criteria included all adults over 18 years old hospitalized at TMAPH in the past year. Patients were consecutively recruited from August 2019-October 2019 and asked to rate aspects of their relationship with their primary care provider (PCP). Descriptive statistics and multivariable logistic regression were used to analyze predictors of the doctor-patient relationship. Patients were interviewed regarding their perceptions of care coordination and doctor-patient relationships. General Thematic Analysis was utilized to analyze qualitative data and develop themes. Quantitative and qualitative findings were then merged to interpret the various dimensions of doctor-patient relationships.

**Results:**

A total of 150 patients (47.3% male) enrolled. Ten patients underwent qualitative interviews. The median patient age was 67 years (IQR 56–76). 112 (74.7%) of patients identified a PCP either at or outside of TMAPH. 89% had diabetes and/or hypertension. Compared to patients without a PCP, having a PCP led to a significantly higher adjusted odds of always spending optimal time with their doctors (aOR 2.7, 95% CI 1.1–6.8, *p* = 0.04), and always receiving clear instructions on managing their medical conditions (aOR 2.5, 95% CI 1.0–6.1, *p* = 0.04). The following themes were developed from patient interviews: (1) patients trusted and respected their PCP believing they were receiving high quality care; and (2) despite perceived fragmentation in care, patients spoke favorably of their relationships with their doctors.

**Conclusions:**

Among a sample of recently hospitalized patients, those with a PCP reported more positive doctor-patient relationships, though rates of dissatisfaction with doctors were still high. Further research and strategies are required to optimize continuity of care and doctor-patient relationships across the entire continuum of outpatient and inpatient care.

## Introduction

The burden of chronic illness is rapidly increasing in India. 60% of all deaths in India are attributed to non-communicable diseases (NCDs) (1). From 1990 to 2016, mortality attributed to diabetes (DM) and cardiovascular disease (CVD) increased by 250 and 215%, respectively (2, 3). The delivery of quality care to address this growing burden of chronic disease remains a persistent challenge in India (4). Doctor-patient relationships are central to any discussion around quality-of-care for patients. Strong doctor-patient relationships have been shown to improve a wide range of health care outcomes including medication adherence, reduced disease co-morbidity, and mortality (5–8).

Researchers and practitioners in India have observed a deterioration in the doctor-patient relationship, driven by complex systemic and social factors (9, 10). In other low- and middle income countries (LMICs) poor doctor-patient communication, high doctor workload, the inability of patients to return to the same doctor to develop longitudinal relationships, and decreased medical service quality were drivers of lower doctor-patient trust (10–13).

Continuity of care and having a consistent primary care provider have also been used as proxies for the strength of doctor-patient relationships. As defined by primary health care experts, relational continuity as used in our study refers to “a therapeutic relationship between a patient and one or more providers that spans various healthcare events and results in accumulated knowledge of the patient and care consistent with the patient's needs.” (14). In other studies, continuity of care has shown to be associated with higher rates of screening of diabetes (DM) and hypertension (HTN) (15), improved physical and mental health (7), and reduced hospitalization, disease-related complications, and mortality in patients with chronic diseases (8, 13).

Anecdotal evidence from Dr. TMA Pai Hospital (TMAPH), Udupi, an urban secondary-level private hospital in Karnataka, India has found that due to steady increases in complex patient populations, physicians are experiencing increasing pressures on their time in the outpatient setting. As a result, care is increasingly fragmented, with a potential to cause adverse outcomes of re-hospitalization, rising costs, and perceived harm to the patient and doctor experiences. There is also a limited understanding of how such changes were impacting doctor-patient relationships and quality of care more broadly. Beyond the immediate relevance of these findings to TMAPH, research on quality of care and improvements to the doctor-patient relationship is urgently needed to address the increased severity, complexity, and need for continuity of patients with diabetes, hypertension and other chronic conditions in India to prevent hospitalizations and adverse healthcare complications.

In this paper, we describe patient experiences, perceptions, and expectations of doctor-patient relationships of patients seeking care at TMAPH.

## Methods

### Study Design

We carried out a cross-sectional, mixed-method needs assessment with two components: (1) quantitative surveys of patients hospitalized or seen in the outpatient setting after recent hospitalization at TMAPH; (2) in-depth qualitative interviews with a subset of patients. Quantitative and qualitative arms of the study were conducted concurrently.

### Settings and Participants

Udupi is a southern district in the state of Karnataka in India with a population of approximately 1.2 million people. About 28% of the population lives in urban areas (16). The literacy rate ranges from 83.9% (rural) to 92.1% (urban). TMAPH is a private, urban, secondary level hospital located in the city of Udupi (population 144,960) in the district of Udupi which offers services in nearly 15 specialties including general medicine and cardiology. The hospital operates under the umbrella academic institution of Manipal Academy of Higher Education (MAHE) and within the referral network, has close ties to community hospitals and an affiliated tertiary level hospital Kasturba Hospital at Manipal. The Manipal healthcare system is a private hospital system that provides discounts and insurance cards for their patient population. The Manipal Arogya card cuts outpatient patient consultation fees by 50% (17).

### Quantitative Methods

#### Participants

Adult patients admitted to the medical wards or presenting post-discharge at the outpatient clinic at TMAPH were recruited for the study. Eligible participants were those with at least one hospitalization in the medical ward at Udupi (including current hospitalization) in the past year. Exclusion criteria included children <18 years old, pregnant women, patients unable to consent due to altered mental status, patients with active tuberculosis, and patients currently in the intensive care unit.

#### Sample Size

We aimed to recruit 150 or an estimated 5% of the annual population hospitalized at Udupi for a representative sample.

#### Study Tool Validation

A survey tool for patients and providers was co-constructed by study investigators and research coordinators at MAHE and the University of Chicago. Trained translators were employed to translate the survey into Kannada. During a pilot phase in July 2019, a sample of five hospitalized patient-participants underwent cognitive interviewing. Surveys were revised accordingly.

#### Data Collection

From August 2019 to October 2019, patients were recruited consecutively. The research team reviewed the list of patients admitted to TMAPH with the medical team to determine which patients were appropriate to recruit and interview based on inclusion and exclusion criteria and proximity to discharge date. Patients were recruited at or within 24 h of discharge to avoid interference with the provision of medical care for active medical issues. The research team also recruited patients from the outpatient clinic at TMAPH. 150 patients consented and completed quantitative surveys. Tablets were used to record survey data into REDCAP. Data from patients were collected on socio-demographics, self-rated health, satisfaction with outpatient care delivery, and outpatient doctor-patient relationships at TMAPH and outside of TMAPH.

Hall et al. (18) outline five key provider qualities necessary to build strong doctor patient relationships, including *fidelity* (genuine interest in a patient), *honesty, competence* (both knowledge and communication skills), *confidentiality, and global trust*. To assess the outpatient patient-doctor relationship, patients were asked to rate their doctors as always, sometimes, or never for the following elements (previously validated at University of Chicago) (19, 20). A primary care provider (PCP) in this study was defined as a qualified health care provider with either a MBBS degree (MD in United States), internal medicine specialization or family medicine specialization who provides continued care (2+ visits) and is the first provider of contact regardless of health concern (e.g., not limited by organ system or type of health concern) for a patient.

Binary outcomes were categorized as optimal (always) and suboptimal (sometimes, never).

- In the past 12 months, how often was this doctor knowledgeable about your medical history?
- How often did you feel that you could tell your doctor anything, even things you might not tell anyone else?
- How often did the doctor explain things in a way that was easy to understand?
- How often did this doctor spend enough time with you?

#### Statistical Analysis

Descriptive statistics were used to assess the quantitative data in this study. Multivariable logistic regression was used to determine the patient reported factors that were associated with components of the doctor-patient relationship including having enough time with their doctors, trusting their doctors, receiving clear instructions from their doctors, and having their doctors always being knowledgeable of their medical conditions. Additional factors included underlying medical conditions, and identifying a PCP. STATA v. 15 was used for quantitative data analysis.

### Qualitative Methods

#### Participants

Maximum variation sampling—a form of purposive sampling—was utilized to identify participation (21). We identified patients in order to construct a sample consisting of patients with a diverse range of characteristics—age, gender, socioeconomic status, and co-morbidities—but who were all hospitalized at least once within the past year (22). Through this sampling approach and after achieving data saturation, we ultimately recruited ten participants from the quantitative survey phase of the study to participate in a qualitative interview. Exclusion criteria included children <18 years old, pregnant women, patients unable to consent due to altered mental status, patients with tuberculosis and patients currently in the intensive care unit.

#### Interview Guide Development and Data Collection

The research team at MAHE and University of Chicago developed the interview guide to elicit responses around health care needs, expectations of the doctor-patient relationship, and experiences in the clinic and hospital. Table 1 outlines a selection of questions from the interview guide. Trained research assistants and members of the research team conducted the interviews. Written informed consent for the quantitative interview included a section on a chance of being selected for a qualitative interview. Interviews were audio-recorded, lasted approximately 30–45 mins and were conducted in Kannada, Tulu, Hindi, and English based on the language preference of the participant. Patient interviews were de-identified, transcribed and translated into English by a contracted transcriber.

**Table 1:** Qualitative interview guide excerpt.

**Interview guide**
**Domain I: patient needs**
Let's start by discussing your healthcare needs.
How would you describe your health right now?
What role does your doctor play in keeping you healthy?
**Domain II: Patient expectations of the provider—patient relationship**
When you are choosing a doctor, what factors are important to you?
How do these factors change when you have a short-term illness, such as a cough or fever? Where do you seek treatment?
What about chronic conditions, such as diabetes or hypertension? Where do you/would you seek treatment?
How would you describe your relationship with your doctor at Dr. TMA Pai Hospital? (Probe: how well do they know you as a person, your medical history, coordinating with other doctors?)

#### Data Analysis

Qualitative data were analyzed using General Thematic Analysis (23). This is an approach that allows for theoretical framework flexibility to arrive at themes that explain people's experiences, perceptions, or representations of a topic. The codebook was developed by VS, CK and PG using an inductive approach, where codes were generated from the data using line-by-line coding with three transcripts. After the codebook was finalized, two analysts (VS, CK) both coded ten patient transcripts manually using Microsoft Word. Analysts discussed reached consensus on points of disagreement through frequent discussions. When all ten transcripts were coded with consensus reached among the coders, one analyst (VS) reviewed the coded data and developed themes by (1) reviewing data from *within* each code in order to understand patterns in patient experiences; (2) by reviewing data *across* codes in order to develop broader themes. Weekly discussions with VS, CK, and PG refined the analysis.

#### Mixed Method Analysis

Data from both strands were triangulated using a framework from Hall et al. on doctor patient relationships. The research team analyzed findings concurrently and merged in order to bolster interpretation of findings. For example, qualitative data were used to illustrate key findings from the quantitative results from the survey data.

### Ethics

The study protocol and all study materials were approved by the institutional review boards of Manipal Academy of Higher Education, Manipal, Karnataka, India, and the University of Chicago, Chicago, IL.

## Results

### Patient Demographics and Co-morbidities

**Figure 1:**
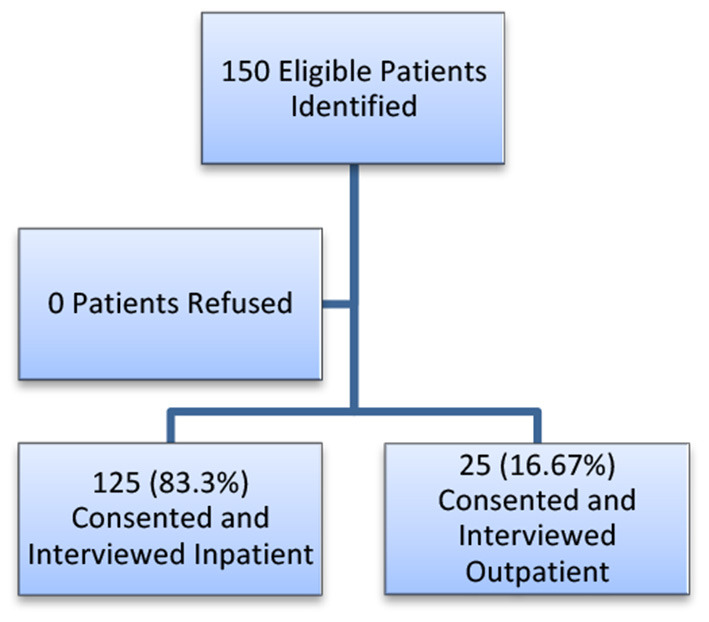
Patients consented and enrolled in the study.

A total of 150 patients were consecutively recruited, 125 (83.3%) were enrolled from the inpatient setting and 25 (16.7%) were enrolled from the outpatient setting post-discharge. All eligible patients approached by research assistants consented and enrolled in the study (Figure 1).

Patients' median age was 67 years (IQR, 56–76 years) and 47% of patients were male. 19.5% of patients completed a high school level of education. 38% held a Below Poverty Line (BPL) card. 12.8% reported being unemployed, not including categories of retired and homemakers. The average number of co-morbidities in this population was 2.4 (SD 1.0). Seventeen (11.3%) patients had no underlying DM/HTN, 41 (27.3%) had HTN alone, 26 (17.3%) had DM alone, and 66 (44%) had both HTN and DM. Patients visited the outpatient clinic an average of 2.1 (SD 1.4) times over the course of 12 months. The average number of hospitalizations in the past 12 months was 1.4 (SD 0.9) and the average number of lifetime hospitalizations was 4.1(SD 4.2) (Table 2). 112 (74.7%) patients reported having a primary care provider, and 38 (25.3%) reported having no primary care provider.

**Table 2:** Socio-demographic factors and co-morbidities of patients by PCP status.

	**Primary care doctor (*n* = 112)**	**No primary care doctor (*n* = 38)**	**Total (*n* = 150)**	***p*** **value**
Age (median)	67 (IQR 53–75.5)	69.5 (IQR 58–76)	67 (IQR 56–76)	
Age over 65	8 (47%)	26 (63%)	88 (59%)	0.07
Gender (male)	51 (46%)	20 (53%)	71 (47%)	0.45
High school diploma	43 (38%)	15 (39%)	58 (39%)	0.91
Unemployed	13 (12%)	6 (16%)	19 (13%)	0.42
Number of co-morbidities	2.3 (1.0)	2.6 (1.1)	2.4 (1.1)	0.18
Below poverty line (BPL) ration card^*^	33 (35%)	15 (44%)	48 (38%)	0.87
Rural residence	41 (37%)	20 (53%)	61 (41%)	0.08
Number of hospitalizations in past 12 months	1.4 (0.9)	1.5 (1.0)	1.4 (0.9)	0.9
Number of lifetime hospitalizations	4.1 (4.3)	4.3 (3.7)	4.1 (4.2)	0.78
Distance to TMA pai hospital (minutes)	30.9 (2.6)	37.4 (7.9)	32.5 (2.8)	0.31
Yearly median household expenditure on healthcare	16,476 INR (1,772 INR)	8,851 INR (2,435 INR)	14,517 INR (1,883 INR)	0.08
Insurance coverage	89 (79%)	31 (84%)	120 (81%)	0.57
**Co-morbidities**				
Number of Co-morbidities	2.3 (1.0)	2.6 (1.1)	2.4 (1.1)	0.18
No HTN/DM	13 (12%)	4 (11%)	17 (11%)	0.86
HTN and/or DM	99 (88%)	34 (90%)	133 (89%)	0.86
Hypertension only	30 (27%)	11 (29%)	41 (27%)	0.80
Diabetes only	23 (21%)	3 (0.1%)	26 (17%)	0.08
HTN/DM combined	46 (41%)	20 (53%)	66 (44%)	0.22

* *Below Poverty Line is used by the Indian government to identify economically disadvantaged households in need of government assistance. The criteria are varied by state and between rural and urban communities*.

Among the ten patients that underwent qualitative interviews, 30% were male and ages ranged from 47–80 years (median 65.5). Most patients were above poverty level (60%), 20% lived in rural areas, 30% in urban areas, 40% in sub-urban areas, and 50% had an education below high school level. Patients had between 2–5 chronic conditions (median 3), 60% with DM, 90% with HTN, 30% with cardiac disease, and had been hospitalized 1-23 (median 5) times in their lifetimes (Supplementary Materials).

### Awareness of Diabetes and Hypertension

When compared to diagnoses listed on a patient's medical chart, 94.4% of patients with HTN were aware of having HTN and 96.8% of those with DM were aware of having DM. In comparison, fewer patients (71.1%) with a diagnosis of cardiac disease (heart failure, ischemic heart disease) in their chart were aware of that diagnosis.

### Hospitalizations

There was no difference in number of hospitalizations in the past 12 months (*p* = 0.78) or number of lifetime hospitalizations (0.31) between those who had a PCP and those who did not (Table 2). Of note, the average number of lifetime hospitalizations was highest in those with co-morbid DM and HTN (No disease: 3.7 vs. HTN: 4.10 vs. DM: 3.16 vs. HTN/DM: 4.6) (Supplemental Materials). The most common chief complaints about last or current hospitalization for all patients included infections (51.2%) followed by an acute exacerbation of chronic illness (36.6%) (Table 3).

**Table 3:** Most common reasons for hospitalization.

Most common reasons for hospitalization (*n* = 123) ^*^	**Percentage**
**Infection total**	**51.2%**
Respiratory infections (*n* = 23)	18.7%
Urinary tract infections (*n* = 13)	10.5%
Dengue, malaria (*n* = 9)	7.3%
Cellulitis (*n* = 8)	6.5%
Gastrointestinal: vomiting, diarrhea (*n* = 5)	4.1%
Other (dengue, non-specific fever, malaria, sinusitis) (*n* = 5)	4.1%
**Exacerbation of chronic disease total**	**37.4%**
COPD/Asthma (*n* = 19)	15.4%
Diabetes/hyperglycemia (*n* = 19)	15.4%
Cardiovascular complications (stroke, heart failure, high blood pressure) (*n* = 8)	6.5%
**Miscellaneous (hemoptysis, anemia, weakness, liver disease, fainting, sodium deficiency) (*****n*** **=** **14)**	**11.4%**

* *27 missing reasons for hospitalization*.

### Characterizing the Doctor-Patient Relationship

**Figure 2:**
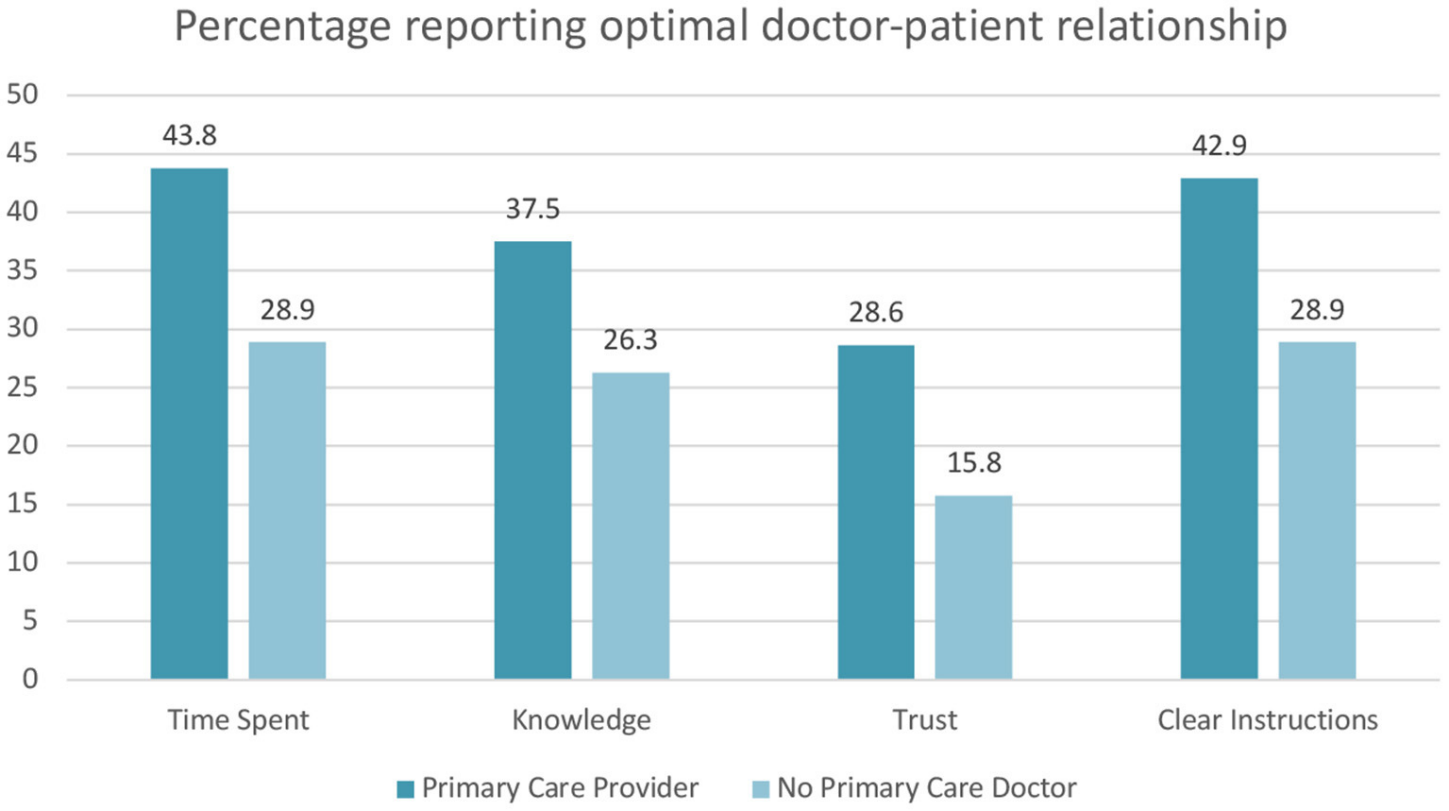
Percentage of patients reporting their doctors always spent enough time with them, always are knowledgeable about their medical history, can always trust with any information, and always provide clear instructions by PCP status.

Compared to patients without a PCP, after controlling for age and gender, having a PCP led to a significantly higher odds of always spending optimal time with their doctors (OR 2.7, 95% CI 1.1–6.8, *p* = 0.04), always trusting their doctor with their medical information (OR 2.7, 95% CI 1.0–7.4, *p* = 0.05), and always receiving clear instructions on managing their medical conditions from their doctors (OR 2.5, 95% CI 1.0–6.1, *p* = 0.04). There was a trend toward significance of a higher odds of those with a PCP always reporting that their doctor was knowledgeable of their medical history (OR 2.3, 95% CI 0.9–5.6, *p* = 0.07) (Table 4; Figure 2). Amongst those with either DM and/or HTN, those with DM alone reported lower odds of always receiving clear instructions about managing their medical conditions (OR 0.2, 95% CI 0.04–0.9, *p* = 0.03) and lower odds of their doctors always being knowledgeable about their medical conditions (OR: 0.2; CI 0.04–0.99; *p* = 0.05) (Figure 2; Table 4). See Supplementary Materials for a breakdown of each component of the doctor-patient relationship by underlying condition (DM, HTN, DM/HTN, neither) and having a PCP.

**Table 4:** Logistic regressions for the perception of the patient-doctor relationship.

**Variables**	Odds ratio—Time spent^*^	***p*** **value**	Odds ratio—Knowledge of patient medical history^*^	***p*** **value**	Odd ratio —Trust^*^	***p*** **value**	Odds ratio—Clear instructions^*^	***p*** **value**
**Age**	1.0 (CI 0.98–1.0)	0.38	1.0 (CI 0.97–1.0)	0.9	1.0 (CI 0.97–1.03)	0.91	1.0 (CI 0.97–1.0)	0.95
**Gender**	0.6 (CI 0.3–1.4)	0.23	1.2 (CI 0.5–2.5)	0.72	1.5 (CI 0.6–3.4)	0.38	1.1 (CI 0.5–2.4)	0.82
**Below poverty line**	0.5 (CI 0.2–1.1)	0.08	0.98 (CI 0.4–2.2)	0.97	0.9 (CI 0.3–1.9)	0.78	1.3 (CI 0.6–2.9)	0.53
**Having a primary care provider**	2.7 (CI 1.1–6.8)	0.04	2.3 (CI 0.9–5.6)	0.07	2.7 (CI 0.997–7.4)	0.051	2.5 (CI 1.0–6.1)	0.04
**Hypertension** ^**+**^	0.2 (CI 0.05–0.8)	0.03	0.7 (CI 0.2–2.8)	0.65	0.7 (CI 0.2–2.7)	0.6	0.5 (CI 0.1–1.9)	0.32
**Diabetes** ^**+**^	0.08 (CI 0.01–0.4)	0.003	0.2 (CI 0.04–0.99)	0.050	0.2 (CI 0.03–1.02)	0.053	0.2 (CI 0.04–0.9)	0.03
**Hypertension and diabetes** ^**+**^	0.3 (CI 0.08–1.3)	0.1	0.8 (CI 0.2–2.9)	0.76	0.8 (CI 0.2–2.8)	0.70	0.8 (CI 0.2–2.9)	0.79

* *95% confidence interval*.

+ *As compared to patients with no diabetes nor hypertension*.

Several patients reported respecting and trusting their PCPs, praising their bedside manner, the time they make for patients no matter how busy they might be, and the way they explain medical conditions.

*An 80-year-old male with DM, HTN, cardiac disease, kidney disease, four lifetime hospitalizations who was retired with a post-graduate education living in an urban setting reported “His [Dr. D] medicines are best. The way Dr. D talks is best and the way this doctor looks after me is best. You cannot find this [quality of care] anywhere.”*

*A 64-year-old F with DM and HTN who lived below poverty in a rural setting noted “Yes, we come here only, as Dr. Z [explains], I follow. I won't go anywhere else, I want him only to see me. He'll also treat me only, however busy he might be...”*

While patients complained about long wait lines to see their doctors, there was no mention of having insufficient time with their doctor during a clinic visit.

### Continuity of Care With Doctors

While many patients identified individual doctors they saw regularly, there was evidence of fragmented care. Some patients saw multiple doctors due to their high burden of chronic diseases and visits to both generalists (MBBS, internal medicine, family medicine) and specialists.

*An 80-year-old M with DM, HTN, cardiac disease, and kidney disease reports: “Yeah sometimes I see other doctors also because I go to Manipal KMC… There I had the chance to see many doctors… There are different types of doctors there.”*

At times discontinuity was due to doctors leaving TMAPH or having long wait times until the next appointment.

*A 67-year-old M with DM, HTN, cardiac disease, thyroid disease, kidney disease, and over 20 lifetime hospitalizations commented on seeing a few different primary care physicians over time. “When I [first] came, Dr. Z was here. When she left from here…they gave me an appointment for 3 months to Dr. A, then Dr. B, now Dr. A or Dr. B.”*

Some patients did not perceive this as an issue; one patient noted that they would be comfortable with any provider, as each doctor “is like a god.” Other patients discuss their preferences for longitudinal care in the hospital and as outpatient with specific doctors that they trust. Some patients insisted on continuity of care with those doctors with skepticism to the quality of care they would receive from providers who were not their PCP. For these patients, their PCP provided them with confidence and reassurance.

*A 73-year-old female with lung disease, HTN, and liver disease, five hospitalizations and a high school education noted, “We come[mainly] to meet him. We don't go to anyone else. When Dr. C tells me or explains, I feel confident about my health. With others, I think I am not sure I will get the same reassurance. That's why [when] I fall [sick], I don't visit any other doctor. Very rarely.”*

### Additional Factors Affecting the Doctor-Patient Relationship

In the quantitative data, there was no significant difference in perceptions of components of the doctor-patient relationship associated with age, gender, or below poverty line (BPL) status (Table 4). In qualitative analyses, some patients more highly educated or with a higher socio-economic status differentiated between specialist physicians and PCPs, but otherwise there were no clear distinctions when patients described their doctors.

*A retired 80-year-old M with a post-graduate education with DM, HTN, cardiac disease, and kidney disease, four lifetime hospitalizations and living APL recalled about his primary care doctor “Though he is not a cardiology doctor, he is quite capable of answering certain questions though [it is not his expertise]*.

At times a combination of the education of a patient, their health awareness, and age resulted in more doctor communication with family members than the patient.

*A 56-year-old F with DM, HTN, and lung disease, two lifetime hospitalizations, and with a BPL card denied discussing her health problems with her doctors:*

“***I:**** About your health problems, do they discuss with you about the disease and how is it?*

***P:**** No, no*.

***I:*** *Say nothing? Do they tell your children?*

***P:*** *Yes, they [tell my] children.”*

Finally, patients that identified a primary care doctor had a trend toward higher yearly median household expenditure on healthcare [16,476 INR (221 USD) vs. 8,851 INR (119 USD), *p* = 0.08]. Insurance coverage was similar in both groups of patients (Table 1). Patients in qualitative interviews commented on out-of-pocket costs of medical care in the private sector but still preferred TMAPH over other facilities.

*A 67-year-old M with five chronic conditions (DM, HTN, cardiac disease, kidney disease, thyroid disease), APL, retired with a higher secondary education living in an urban setting reported compliance with his medications despite it being financially difficult to cover all expenses with his insurance health card, he recognizes the importance of managing for his health. “if it is costly, no problem, [my] health is first.”*

*He continues: “Once I asked here, is there any little low-cost facility…?” …Dr. Y told me, “There is one but I won't advise you [to go there].”… I thought taking some 20% discount losing my health is not a good choice so I canceled that one.”*

## Discussion

There has been a steady rise in the numbers of patients with complex needs (i.e., patients living with multiple chronic conditions) in India. One sub-national study found that nearly a third of patients utilizing primary care presented with multi-morbidities (24). Doctor-patient relationships in the outpatient setting, longitudinal continuity of care, and high-quality care are necessary for adequate disease control for these chronic disease patients.

Our study of a population with high rates of DM and/or HTN showed that having a PCP was associated with a higher odds ratio of patients reporting optimal doctor-patient relationships compared to not having a PCP. Patients highly praised doctors that spent adequate time with them, communicated effectively, and whom they trusted with confidential personal information. However, there were still major gaps. Notably even with a PCP, less than half of this population reported always spending adequate time with their doctors, always receiving clear instructions from their doctors, and only 28.6% of those with a PCP reported always trusting their provider with medical and personal information (Figure 2). Having a PCP alone may not be as important as developing doctor-patient relationships built on confidentiality, global trust, fidelity, honesty, communication and medical knowledge competence (18) with any one or multiple doctors involved in a patient's care. In the Indian healthcare system, patients seek out primary care providers for general healthcare concerns as well as prevention (e.g., vaccination). The supply of primary care doctors (and qualifications) is dependent on providers choosing general medicine, internal medicine, or family medicine as their specialty, similar to what exists in many other countries worldwide. More research is needed in India to explore the associations between having a PCP, elements of strong doctor-patient relationships and health outcomes.

Despite low rates of trust, receiving clear instructions, and spending adequate time with PCPs noted in quantitative surveys, patients in this study may have demonstrated a social desirability bias in qualitative interviews. When asked to expand on perceptions of doctors, this patient population may have had a tendency to answer more favorably or positively for multiple reasons. Patients were interviewed in the healthcare setting, which may not have felt like a secure, objective environment for all patients. To counter this, all surveys and interviews were conducted in private settings by research staff not associated with the hospital or outpatient clinic. Additionally, inpatient interviews were conducted on or 24 h prior to the day of discharge to eliminate any fear that participation and their responses would jeopardize their clinical care. This patient population also actively chose to seek care at TMAPH instead of going to local or public facilities due to inherently favorable perceptions of TMAPH.

Our quantitative study showed no difference by age, gender, and below poverty line status on perceptions of the doctor-patient relationship. Our qualitative data suggested that age, gender, education, complexity of medical disease may all impact how doctors interact with patients and a patient's perception of their doctor, which is similar to other studies (25, 26). There are many factors that may explain this discrepancy including the wording and patient understanding of quantitative vs. qualitative questions. The categorical questions in the patient survey may have been insufficient in capturing the nuance of patient perceptions of their doctors, which in this study population seemed to encompass not only how doctors communicate with patients but also how doctors incorporate and communicate with patients' families.

Currently, there is a push to improve quality of care in primary care in LMICs, especially with growing rates of DM, HTN and chronic diseases (27–29). While quality has been measured using the cascade of care of care (30–33) and achievement of guideline-based management and counseling strategies (34, 35), fewer studies in LMICs include the role of longitudinal primary care doctors, empanelment, and the doctor-patient relationship in quality-of-care assessments (36). Given that research has shown that continuity of care improves medication adherence and patient healthy lifestyle behaviors (37–39), more research is needed on the state of continuity of care in LMICs and the doctor-patient relationship. Interventions targeting strengthening this critical relationship and continuity of care with a PCP need to be tested and evaluated in LMICs (36).

Strengths of this study included a mixed methods methodology and a focus on patient perceptions of their experience of the healthcare system. The adequate management of DM and HTN requires patient activation, autonomy and empowerment. Better understanding the factors that affect patient perceptions and patient empowerment is necessary to designing interventions to better manage DM and HTN.

Limitations to this study include due to timeline and convenience, the recruitment of a larger portion of the study population in the hospital instead of the outpatient clinic after discharge. Given this small sample size, we were unable to evaluate the effect of different locations of recruitment on patient perceptions. Patients recruited in the hospital may have more recall bias regarding their outpatient experiences than patients recruited in the outpatient setting.

Our study had a small sample size of patients reporting no PCP. Our study was not designed to evaluate the relationship between perception of the doctor patient relationship, having a PCP and healthcare outcomes. A larger study is recommended to test the hypothesis that a having a PCP improves the doctor-patient relationship as suggested by this study. In the coming year we plan to implement a comprehensive care program at TMAPH to address some of the barriers we identified in this study and determine if strengthening the doctor-patient relationship in India leads to improved health outcomes in medically complex, chronic disease patients.

Our study is one of few studies in LMICs highlighting the association between having a primary care doctor and the doctor-patient relationship in the context of chronic disease (9, 40, 41) and more research is needed to characterize the facilitators and barriers to strong doctor-patient relationships more broadly. There is an urgent need for better disease control amongst HTN and DM patients in India.

Interventions to date have had inadequate impact and reach and there is a dire need to better understand and strengthen the doctor-patient relationship and continuity of care in India.

## Data Availability Statement

The raw data supporting the conclusions of this article will be made available by the authors, without undue reservation.

## Ethics Statement

The studies involving human participants were reviewed and approved by University of Chicago, Manipal Academy of Higher Education. The patients/participants provided their written informed consent to participate in this study.

## Author Contributions

PG, VS, KB, EP, SU, and DM were all involved in designing the study. CK was the study coordinator and helped pilot test, edited and revised the surveys, and along with PG and VS developed an implementation strategy. PG analyzed the quantitative data, wrote the first draft of the manuscript with additions from CK and VS, and finalized the manuscript incorporating edits from VS, KB, EP, SU, RV, and DM. CK and VS analyzed the qualitative data. All authors contributed to the article and approved the submitted version.

## Funding

This project was funded by the Section for Hospital Medicine, Department of Medicine, University of Chicago.

## Conflict of Interest

The authors declare that the research was conducted in the absence of any commercial or financial relationships that could be construed as a potential conflict of interest.

## Publisher's Note

All claims expressed in this article are solely those of the authors and do not necessarily represent those of their affiliated organizations, or those of the publisher, the editors and the reviewers. Any product that may be evaluated in this article, or claim that may be made by its manufacturer, is not guaranteed or endorsed by the publisher.
