# Supplementary material for: Perceptions of the Doctor-Patient Relationship Among Patients in a Private, Secondary-Level Hospital in Southern India
Source: Front Public Health. 2022 Apr 6;9:768705. doi: 10.3389/fpubh.2021.768705 (PMC9019150; doi:10.3389/fpubh.2021.768705)
Supplement: Supplementary file 1 [file Data_Sheet_1.docx]

Supplementary Material

# Supplementary Figures and Tables

| **Domain I: Patient Needs**  Let’s start by discussing your healthcare needs.   1. How would you describe your health right now? 2. What role does your doctor play in keeping you healthy? 3. What are some things that you do on your own to stay healthy? 4. Do you believe that the medical conditions that you currently have will continue for a long time? [Prompts]    1. For how long?    2. Please explain your answer. |
| --- |
| **Domain II: Patient Expectations of the Physician – Patient Relationship**   1. When you are choosing a doctor, what factors are important to you? 2. How do these factors change when you have a short-term illness, such as a cough or fever? Where do you seek treatment? 3. What about chronic conditions, such as diabetes or hypertension? Where do you/would you seek treatment? 4. How would you describe your relationship with your doctor at Dr. TMA Pai Hospital? (Probe: how well do they know you as a person, your medical history, coordinating with other doctors?) 5. What are some reasons why you come to Dr. TMA Pai Hospital? 6. How would you feel if you were assigned to see a postgraduate in general medicine for your care? 7. What about a community medicine specialist? 8. What about an MBBS graduate? 9. Would your views change if you knew that any of these doctors were working in a team with a specialist? Why or why not? |
| **Domain III: Patient Experiences in the Clinic and Hospital**  1. How would you describe your experience visiting the outpatient clinic at Dr. TMA Pai Hospital?   - What has been good about your experience? - What can be improved?   2. How would you describe your experience as a patient admitted to the hospital Dr. TMA Pai Hospital?   - What has been good about your experience? - What can be improved?   3. Do you have any concerns about paying for your care at this hospital?  4. Have you ever been referred to or transferred to another hospital? If so, please share the reasons for why you were referred and/or transferred.  5. Is there anything else that you would like to tell us about your health care? |

Table 1: Full Qualitative interview guide

| Patient | Age | Sex | Number of chronic conditions | Chronic Conditions | Lifetime Hospital Admissions | Poverty Line* | Type of Insurance | Education | Home Setting | Occupation |
| --- | --- | --- | --- | --- | --- | --- | --- | --- | --- | --- |
| 1 | 80 | M | 4 | DM, HTN, cardiac and kidney disease | 4 | APL | LIC Insurance | Post-Graduate | Urban | Retired |
| 2 | 53 | F | 3 | DM, HTN, lung disease | 3 | APL | ICICI insurance | Graduate | Urban | Self-employed |
| 3 | 78 | F | 3 | HTN, cardiac disease, cancer | 6 | APL | No insurance | Literate | Rural | Unemployed |
| 4 | 73 | F | 2 | Lung and lung disease | 5 | APL | no insurance | High school certificate | Urban | Homemaker |
| 5 | 64 | F | 2 | DM, HTN | 10 | BPL | Manipal Arogya | Middle school certificate | Rural | Self-employed |
| 6 | 67 | M | 5 | DM, HTN, cardiac, kidney and thyroid disease | 23 | APL | unknown | Higher secondary certificate | Urban | Retired |
| 7 | 52 | M | 2 | HTN, liver disease | 2 | APL | Manipal Arogya | Graduate | Sub-urban | Government employee |
| 8 | 67 | F | 2 | HTN, lung disease | 5 | BPL | Manipal Arogya | Literate | Sub-urban | Homemaker |
| 9 | 56 | F | 3 | DM, HTN, lung disease | 2 | BPL | Manipal Arogya | Literate | Sub-urban | Homemaker |
| 10 | 47 | F | 2 | DM, HTN | 1 | BPL | Manipal Arogya | Literate | Sub-urban | Daily wage |
| Table 2: Socio-demographics of patients undergoing qualitative interviews | | | | | | | | | | |

|  | No Diabetes or Hypertension (n=17) | Hypertension (n=41) | Diabetes (n=26) | Diabetes and Hypertension (n=66) | Total  (n=150) |
| --- | --- | --- | --- | --- | --- |
| Age (median) | 60 (IQR 20) (IQR Q1, Q3) | 71 (IQR 21) | 56.5(IQR 23) | 69(IQR 16) | 67 (IQR 56-76) |
| Age over 65 | 8(47%) | 26(63%) | 9(35%) | 45(68%) | 88(59%) |
| Gender (Male) | 8(47%) | 18(44%) | 11(42.3%) | 34(51.5%) | 71(47%) |
| High School Diploma | 4(23.5%) | 6(14.6%) | 7(28%) | 12(18.2%) | 29(19.5%) |
| Unemployed | 0 | 8(19.5%) | 4(15.4%) | 7(10.6%) | 19(12.8%) |
| Number of Co-Morbidities | 1.2 (0.4) | 2.2 (0.8) | 1.7 (.9) | 3.1 (1.0) | 2.4 (1.0) |
| Below Poverty Line (BPL) Ration Card* | 6(46%) | 10(27.8%) | 11(47.8%) | 21(38%) | 48(38%) |
| Rural Residence | 5(29%) | 20(48.8%) | 10(38.5%) | 26(39.4%) | 61(40.7%) |
| Number of Hospitalizations in Past 12 Months | 1.3 (0.6) | 1.4 (1.1) | 1.4(0.6) | 1.5(0.9) | 1.4(0.9) |
| Number of Lifetime Hospitalizations | 3.7(2.69) | 4.1(3.97) | 3.16(2.64) | 4.61(4.99) | 4.1(4.2) |
| Distance to TMA Pai Hospital (minutes) | 45.9(40.7) | 35.5(32.3) | 31.6(56.3) | 27.6(17.2) | 32.5(33.9) |
| Yearly Median Household Expenditure on HealthCare | 19,764 INR | 19,653 INR | 8,999 INR | 15,046 INR | 15,796 INR |
| Insurance Coverage | 10(59%) | 34(85%) | 19(73%) | 57(85.4%) | 120(80.5%) |
| Primary Care Doctor for Chronic Disease Care | 13(76.5%) | 30(73.2%) | 23(88.5%) | 46(69.7%) | 112(74.7%) |
| Number of Outpatient Visits in last 12 months | 1.9(1.3) | 1.8(1.3) | 1.8(1.1) | 2.4(1.5) | 2.1(1.4) |
| Table 3: Socio-demographic factors and co-morbidities of patients by DM/HTN Status | | | | | |

Figure 1: Percentage of patients reporting optimal doctor-patient relationships by DM/HTN status

|  | **PCP** | **No PCP** | **PCP** | **No PCP** | **PCP** | **No PCP** | **PCP** | **No PCP** | **PCP** | **No PCP** | **PCP** | **No PCP** |
| --- | --- | --- | --- | --- | --- | --- | --- | --- | --- | --- | --- | --- |
| **Co-Morbidities** | *Neither* | *Neither* | *HTN* | *HTN* | *DM* | *DM* | *HTN/*  *DM* | *HTN/*  *DM* | *Total **  *(HTN and/or DM)* | *Total**  *(HTN and/or DM)* | *Total*** | *Total*** |
| **Time Spent** | 9 (69%) | 1(25%) | 14(47%) | 2(18%) | 3(13%) | 0(0%) | 22(48%) | 8(40%) | 39(39%) | 10(29%) | 49(44%) | 11(29%) |
| **Patient History Knowledge** | 5(38%) | 1(25%) | 15(50%) | 1(9%) | 3(13%) | 1(33%) | 18(39%) | 7(35%) | 36(36%) | 10(29%) | 42(38%) | 10(26%) |
| **Trust** | 3(62%) | 1(25%) | 12(40%) | 0(0%) | 2(8.9%) | 1(33%) | 14(30%) | 4(20%) | 28(28%) | 5(15%) | 32(29%) | 6(16%) |
| **Clear Instructions** | 8(62%) | 1(25%) | 13(43%) | 1(9%) | 4(17%) | 1(33%) | 22(48%) | 8(40%) | 39(39%) | 10(29%) | 48(43%) | 11(29%) |
| **Total** | 13(76%) | 4(24%) | 30(73%) | 11(27%) | 23(88%) | 3(22%) | 46(74%) | 20(26%) | 99(74%) | 34(26%) | 112(75%) | 38(25%) |

*No statistically significant difference between having a PCP and not having a PCP on time spent, knowledge of the history of patient’s medical conditions, trust, clear instructions for patients with DM and/or HTN

** No statistically significant difference between having a PCP and not having a PCP on time spent, knowledge of the history of patient’s medical conditions, trust, clear instructions in all patients

Table 4: Perception of patient-doctor relationship by underlying co-morbidities and having a PCP
